# Development of a Mathematical Model for Predicting the Average Molten Zone Thickness of HDPE Pipes During Butt Fusion Welding

**DOI:** 10.3390/polym17141932

**Published:** 2025-07-14

**Authors:** Donghu Zeng, Maksym Iurzhenko, Valeriy Demchenko

**Affiliations:** Department of Plastics Welding, E.O. Paton Electric Welding Institute of the National Academy of Sciences of Ukraine, 03150 Kyiv, Ukraine; dvaleriyl@ukr.net

**Keywords:** HDPE pipe, mathematical model, CFD model, molten zone thickness, butt fusion welding

## Abstract

Currently, the determination of the molten zone thickness in HDPE pipes during butt fusion welding primarily depends on experimental and numerical methods, leading to high costs and reduced efficiency. In this study, a mathematical (MM) model based on Neumann’s solution for the melting of a semi-infinite region was developed to efficiently predict the average molten zone (AMZ) thickness of HDPE pipes under varying heating temperatures and heating times while incorporating the effects of heat convection. Additionally, a two-dimensional CFD model was constructed using finite element analysis (FEA) to validate the MM model. Welding pressure was not considered in this study. The effects of heating temperature, heating time, and heat convection on the AMZ thickness in HDPE pipes were systematically analyzed. The heating temperature at the heated end of HDPE ranged from 190 °C to 350 °C in 20 °C increments, with a temperature of 28 °C as the ambient and initial setting, and the heating time was set to 180 s for both the MM and CFD models. The results demonstrate a strong correlation between the AMZ thickness predictions from the MM and CFD models. The relative error between the MM and CFD models ranges from 0.280% to 10,830% with heat convection and from −2.398% to 8.992% without heat convection. Additionally, for the MM model, the relative error between cases with and without heat convection ranges from 0.243% to 0.433%, whereas for the CFD model, it varies between 1.751% and 3.189%. These findings confirm the reliability of the MM model developed in this study and indicate that thermal convection has a minimal impact on AMZ thickness prediction for large-diameter, thick-walled HDPE pipes.

## 1. Introduction

High-density polyethylene (HDPE) pipes have been extensively used in infrastructure projects, industrial fluid handling, sewage systems, nuclear power plants, and city gas and water distribution networks, as well as various other critical industrial applications, for decades due to their exceptional material properties and performance advantages [1,2]. HDPE pipes are widely recognized and utilized worldwide due to their numerous advantages, including a highly stable chemical structure, excellent mechanical properties, long service life, superior corrosion resistance, cost-effectiveness, environmental sustainability, and ease of installation [3,4]. A mountain of empirical studies and industrial applications have consistently demonstrated that HDPE pipes outperform traditional metal counterparts in flexibility, abrasion and fatigue resistance, and adaptability to dynamic loads, making them particularly suitable for seismic-prone regions and demanding operational environments [5]. To ensure the watertightness and integrity of piping systems, HDPE pipes are typically joined using butt fusion and electrofusion welding [6]. For large-diameter, thick-walled HDPE pipes, butt fusion welding is the most effective method for ensuring a secure connection [7,8]. The quality of welded joints in HDPE pipes is crucial to their performance and reliability in aforementioned industrial applications [9]. However, in process of butt fusion welding, common defects that may occur due to improper welding conditions include uneven weld beads, cold welds, porosity, carbonization, and cold joints. The characteristics of high-quality welded joints of HDPE pipes, which are primarily influenced by heating temperature [10,11], compression pressure [12], and process time [13], include strength, a leak-free seal, and longevity. The behavior of the molten zone in HDPE pipes can be used to evaluate welding parameters for butt fusion welding [14]. The morphology of the molten zone in HDPE pipes during butt fusion welding is a critical factor that directly influences the overall quality of welded joints. This study uses the molten zone thickness of HDPE pipes in butt fusion welding as the basis for further investigation. Despite advancements in butt fusion welding for large-diameter, thick-walled HDPE pipes, precisely controlling the molten zone thickness of HDPE pipes remains a significant challenge due to the complex thermal and mechanical interactions involved. Existing studies have examined various aspects of molten zone behavior in HDPE pipes, but there is still a lack of accurate mathematical models for predicting molten zone thickness variations based on heating temperature and heating time. Addressing this gap is essential for enhancing the reliability and efficiency of HDPE pipes in butt fusion welding. Therefore, this study aims to address specific research questions by (RQ1) examining the evolution of molten zone thickness under varying heating temperatures and heating times, (RQ2) exploring the impact of heat convection between the surfaces of HDPE pipes and the open environment on AMZ thickness, (RQ3) developing a mathematical model to reliably predict molten zone thickness during butt fusion welding, and (RQ4) analyzing the potential error effects on the molten zone thickness of HDPE pipes. Numerous researchers, both domestically and internationally, have studied the thermal and mechanical behavior of molten zones in HDPE pipes during butt fusion welding, making significant advancements in understanding and application. Niou et al. [15] analyzed real-time changes in molten zone distance by recording and comparing boundary ultrasonic signals using an experimental method. Walid et al. [16] experimentally investigated the effects of heating temperature, heating duration, and applied force on the HDPE welding process and used the results to develop mathematical models linking input parameters to outputs such as temperature distribution and molten polymer thickness. Belaziz et al. [17] examined the microstructural and mechanical properties of weld bead zones in HDPE pipes, with a particular focus on the effects of welding parameters on the molten zone. Lai et al. [6] emphasized that improper molten zone control can cause defects that significantly shorten the pipe’s creep life, highlighting the need for precise welding parameter control during the melting phase. Wang et al. [18] evaluated various welding processes and their impact on the molten zone of thick-walled HDPE pipes. Walid et al. [19] also found that controlling the temperature and thickness of the molten zone can partially improve the technical quality of the welded joint, enhancing overall performance. Shaheer et al. [20] investigated the micro-mechanical properties of the molten zone in butt-fusion-welded HDPE joints using the nanoindentation technique. Although these studies offer valuable and reliable insights [21], they have significant limitations. Many existing approaches rely heavily on experimental and numerical methods, resulting in high experimental and computational costs, which hinder the accurate determination of molten zone thickness in HDPE pipes. Moreover, limited attention has been given to developing more accurate mathematical (MM) models for predicting molten zone thickness, particularly by incorporating heating temperature, heating time, and heat convection as key influencing factors. This gap underscores the need for more accurate MM models that can effectively predict molten zone thickness in HDPE pipes during butt fusion welding while reducing computational and experimental costs.

To bridge this gap, this study presents a novel MM model based on Neumann’s solution for the melting of a semi-infinite region to predict the average molten zone (AMZ) thickness in HDPE pipes during butt fusion welding. The MM model comprises two heat equations for temperature distribution in solid and molten regions, an energy conservation equation, and a temperature continuity equation at the solid–molten interface, complemented by thermal boundary conditions and error functions. This study neglects welding pressure in butt fusion welding and the viscoelastic nature of HDPE pipes, assuming purely conductive heat transfer. Unlike previous studies, this model explicitly accounts for the evolution of AMZ thickness in response to variations in heating temperature, processing time, and heat convection between the surfaces of HDPE pipes and the open environment. While numerical and experimental approaches have been employed in prior studies, the exploration of MM models with predictive capabilities remains limited. The MM model proposed in this study aims to bridge this gap by providing a more accessible and computationally efficient alternative to conventional experimental and numerical methods. Additionally, to validate the reliability of the MM model, a two-dimensional computational fluid dynamics (CFD) model is developed using finite element analysis (FEA). In both MM and CFD models, the AMZ thickness of HDPE pipes is defined as the average distance from multiple solidus–liquidus points on the solid–molten interface to the heated end of the pipe. This study systematically investigates the effects of heating temperature, heating time, and heat convection between HDPE pipe surfaces and the open environment on AMZ thickness during butt fusion welding. In this study, the heating temperature ranges from 190 °C to 350 °C in 20 °C increments, staying below the heat cracking temperature, while the heating time varies from 0 to 180 s in 10 s increments for both the MM and CFD models. The material properties of HDPE pipes involved in the MM and CFD models are benchmarked from the same existing literature.

This study contributes to the field of butt fusion welding of HDPE pipes by developing an MM model for predicting AMZ thickness, which enhances the understanding of the butt fusion welding process. The MM model provides a cost-effective alternative to experimental and numerical methods by reducing reliance on extensive physical testing and high computational costs. The findings of this study offer practical guidance for optimizing welding parameters in butt fusion welding of HDPE pipes, improving the quality of welded joints, and extending the service life of HDPE pipes. Moreover, this study is novel in developing an MM model that explicitly considers the presence or absence of heat convection between the surfaces of HDPE pipes and the open environment, a factor that has not been previously explored in detail within HDPE pipe welding research.

## 2. Materials and Methods

### 2.1. Nonlinear Material Properties of HDPE Pipe

Polyethylene (PE) resin is synthesized by polymerizing ethylene monomers. Depending on polymerization conditions such as pressure and temperature, PE pipes are categorized into five international grades: PE32, PE40, PE63, PE80, and PE100. Among these, PE80 and PE100 are the most widely used in industrial applications. The high-density polyethylene involved in this study [22], a semi-crystalline polymer composite material with a crystallinity ranging from 80% to 90%, is polymerized under a pressure of 1.4 MPa and a temperature of 100 °C. On top of that, HDPE exhibits higher density and crystallinity due to its relatively high molecular mass and the presence of fewer and shorter branch chains. Density and crystallinity influence properties such as hardness, melting point, tensile strength, and stiffness, which are generally proportional to each other. Nevertheless, research on the material properties of HDPE pipes remains insufficient at present. Specifically, the limited studies on HDPE pipes and the complexity of the composite material result in incomplete physical parameter data, particularly in the high-temperature zone above the softening temperature of 80 °C. Moreover, the material properties of HDPE pipes are influenced by various factors, including raw material composition, the polymerization process, environmental conditions, service conditions, aging, and degradation, as well as testing methods and standards. It is well known that the density, thermal conductivity, specific heat capacity, and enthalpy of HDPE pipes employed vary with temperature. Thus, to ensure that the nonlinear material properties of HDPE pipes used in this study are both reasonable and universally applicable in MM and CFD models, typical thermal physical properties are sourced from the existing literature. If the thermophysical material properties at a certain temperature point are missing, interpolation and extrapolation methods can be used to estimate them.

Woo et al. [23] investigated the variations in the thermal physical properties of HDPE over heating time and derived fitting functions for density as a function of temperature, expressed as follows:

(1) $$\left\{\begin{array}{l} \rho =\frac{1}{1.05e^{0.00136T}}\;T\leq 135\;{}^{\circ}C\\ \rho =\frac{1}{1.14+0.0009T}\;T>135\;{}^{\circ}C \end{array}\right.$$

and the thermal conductivity, specific heat capacity, and enthalpy of HDPE with respect to temperature changes, as shown in Table 1. The thermophysical properties of HDPE pipes, as mentioned in Equation (1) and Table 1, will be used in the CFD model using a defining function and interpolation.

In the MM model, this study considers only the thermophysical properties of HDPE pipes in the solid and molten phases, without accounting for the effect of temperature changes on the thermophysical properties of the HDPE pipes, as shown in Table 2. The convective heat transfer coefficient was set to be 7.22 W/(m^2^∙°C) for the outer surface and the open environment and 0.89 W/(m^2^∙°C) for the inner surface of the HDPE pipe and air interlayer, referring to the existing literature.

Studies [26,27] using thermogravimetric analysis (TGA) found that HDPE begins to degrade at approximately 323 °C, with rapid degradation occurring between 384 °C and 500 °C. This serves as a key basis for selecting the heating temperature in this study. Considering the material properties, reaction kinetics, and butt fusion welding process of HDPE pipes, the heating temperature range was set from 190 °C to 350 °C in 20 °C increments to ensure a comprehensive, efficient, and cost-effective investigation.

### 2.2. Mathematical Model for AMZ Thickness of HDPE Pipe

Butt fusion welding joins two sections of HDPE pipe through bead-up, heat soaking, dwelling, jointing, and cooling to form a strong connection [10]. This process takes advantage of the thermoplastic nature of HDPE pipes, which soften around 80 °C and melt near their melting point. When the ends of HDPE pipes are heated to a molten state and pressed together, their molecular chains diffuse, fuse, and cool to solidify the joint. In butt fusion welding, the heat soaking stage is one of the most critical phases, as it ensures the molten zone of the HDPE pipe reaches an appropriate thickness by controlling the heating temperature and heating time. The quality of welded joints in HDPE pipes primarily depends on the molten zone thickness, which determines the proper bead height according to welding standards. Thus, determining the optimal molten zone thickness is crucial for achieving high-quality welded joints in HDPE pipes. Traditionally, molten zone thickness in HDPE butt fusion welding has been determined primarily through experiments and numerical solutions, leading to low welding efficiency and inconsistent quality due to extensive testing for optimal welding parameters.

**Figure 1:**
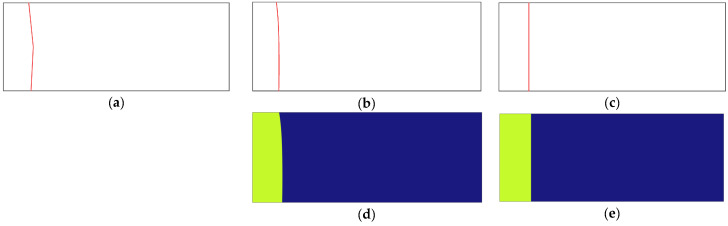
Calculation scheme of AMZ thickness of HDPE pipe in MM and CFD models. (**a**) Scheme of MM model with heat convection; (**b**) scheme of CFD model with heat convection; (**c**) scheme of MM and CFD models without heat convection; (**d**) CFD contour with heat convection; (**e**) CFD contour without heat convection.

Building upon previous research, this study further explores the behavior of the molten zone of HDPE pipes during butt fusion welding. The purpose of this study is to develop an MM model using Neumann’s solution for the melting of a semi-infinite region for predicting the AMZ thickness of HDPE pipe as the heating time progresses, considering cases with and without heat convection between surfaces of HDPE pipes and the open environment. For the development of the MM model, some assumptions need to be made in this study: (1) the thermal physical properties of each phase are uniform and remain constant, (2) there is a negligible effect of liquid phase motion due to changes in density, (3) there is one-dimensional conduction, (4) there is no energy generation, and (5) the density at solid–molten interface is kept as the average density between solid and molten phase. In the MM model, the AMZ thickness of the HDPE pipe was defined as the average of the molten zone thicknesses measured from the triple point on the outer surface to the heated end, from the solidus–liquidus point on the central line to the heated end, and from the triple point on the inner surface to the heated end, as shown in Figure 1a. Heat convection is not considered when determining the molten zone thickness of the HDPE pipes at the central line.

Two heat-governing equations for solid and molten regions are given by Equation (2)

(2) $$\frac{\partial T_{m}}{\partial t}=\alpha_{m}\frac{\partial^{2}T_{m}}{\partial x_{m}^{2}};\frac{\partial T_{s}}{\partial t}=\alpha_{s}\frac{\partial^{2}T_{s}}{\partial x_{s}^{2}}$$

where $\alpha_{m}$, $\alpha_{s}$ denote thermal diffusivity of HDPE pipe in the molten and solid regions.

The heat convection between the surfaces of HDPE pipes and the environment can be expressed by the following equation:

(3) $$-k\frac{\partial T}{\partial x}=h_{\;o\&i}(T-T_{0})$$

where $h_{o\&i}$ is used as the convective heat transfer coefficient, the subscript *o* denotes the convective heat transfer coefficient between the outer surface of HDPE pipes and the open environment, and the subscript *i* denotes the convective heat transfer coefficient between the inner surface of HDPE pipes and the air interlayer.

In the process of butt fusion welding of HDPE pipes, boundary and initial conditions are given by

(4) $$T(x,0)=T_{s}=T_{0};\;\;\;\;T(0,t)=T_{m}=T_{g};\;\;\;\;T(\infty ,t)=T_{s}=T_{0}$$

where $T_{0}$, $T_{m}$, and $T_{g}$ represent the initial temperature, melting point, and heating temperature, respectively, in °C.

As heating time increases, the location of the solid–molten interface moves toward the non-heated end. The energy conservation equation at the solid–molten interface can be described both with and without heat convection between the surfaces of HDPE pipes and the open environment by following equation:

(5) $$k_{m}\frac{\partial T_{m}(x_{m},t)}{\partial n}\left|{\,}_{x_{m}=X_{m}}\right.-k_{s}\frac{\partial T_{s}(x_{s},t)}{\partial n}\left|{\,}_{x_{s}=X_{s}}\right.=\frac{\rho_{m}+\rho_{s}}{2}L\frac{dS}{dt}-h_{o\&i}(T_{s}(x_{s},t)-T_{0})\;k_{m}\frac{\partial T_{m}(x_{m},t)}{\partial n}\left|{\,}_{x_{m}=X_{m}}\right.-k_{s}\frac{\partial T_{s}(x_{s},t)}{\partial n}\left|{\,}_{x_{s}=X_{s}}\right.=\frac{\rho_{m}+\rho_{s}}{2}L\frac{dS}{dt}$$

where $k_{m}$, $k_{s}$ denote the thermal conductivity, W/m·°C, and $\rho_{m}$, $\rho_{s}$ denote the density, $kg/m^{3}$, of HDPE pipes in the molten and solid regions, and L is the latent heat of fusion of HDPE pipes from the solid to molten phase, KJ/kg.

The continuity of temperature at the solid–molten interface should be carefully considered. At the solid–molten interface, the temperatures of both the solid and molten phases are seen to be the same, equal to the melting point of HDPE pipes. The mathematical description of the continuity of temperature at the solid–molten interface is given as follows:

(6) $$T_{m}(x_{m},t)\left|{\,}_{x_{m}=X_{m}}\right.=T_{s}(x_{s},t)\left|{\,}_{x_{s}=X_{s}}\right.=T_{m}$$

According to the law of conservation of mass, it is assumed that the volume of the solid phase decreases by $∆V_{s}$ per unit time while the volume of the molten phase increases by $∆V_{m}$ per unit time, both at the same unit width. Thus, the mass conservation equation in this study can be expressed as

(7) $$\rho_{s}\Delta V_{s}=\rho_{m}\Delta V_{m}$$

The above mass conservation equation can then be derived as

(8) $$\rho_{s}X_{s}\cdot 1=\rho_{m}X_{m}\cdot 1\;\to \;\rho_{s}/\rho_{m}=X_{m}/X_{s}=\beta$$

where $∆V_{s}$, $∆V_{m}$ and $X_{s}$, $X_{m}$ represent the volume (${mm}^{3}$) and line length (mm) of the solid phase reduced and the volume and line length of the molten phase increased during the melting of the HDPE pipes per unit time, respectively. Because the densities of the solid phase and the molten phase in the transition zone are equal, $∆V_{s}$ equals $∆V_{m}$, and $X_{s}$ equals $X_{m}$.

The $X_{m}$ and $X_{s}$ at the solid–molten interface boundary must be proportional to the square root of time because this reflects the natural mathematical properties of the error function (*erf*) and the complementary error function (*erfc*) in describing the diffusion and heat transfer process, illustrating the law of the diffusion front advancing with time. Thus, the relationship between $X_{m}$, $X_{s}$ and heat soaking time is determined by

(9) $$X_{s}=K\sqrt{t};X_{m}=\beta K\sqrt{t}$$

The MM model for predicting the location of the solid–molten interface of HDPE pipes was developed by using the error function

(10) $$T_{m}(x_{m},t)=T_{g}+Aerf(\frac{x_{m}(t)}{2\sqrt{\alpha_{m}t}});\;\;\;T_{s}(x_{s},t)=T_{0}+Berfc(\frac{x_{s}(t)}{2\sqrt{\alpha_{s}t}})$$

where $x_{m}$, $x_{s}$ denote the length of the molten zone and the solid zone of HDPE pipe, respectively, in mm, and t represents the heating time during butt fusion welding in s.

The first equation of Equation (10) can be used to describe the relationship between the molten zone thickness of HDPE pipes and heating time if the value in the left term is given.

By combining Equations (6)–(9), the coefficients *A* and *B* of Equation (10) can be solved as

(11) $$A=\frac{T_{m}-T_{g}}{erf(K\beta /2\sqrt{\alpha_{m}t})};\;\;\;B=\frac{T_{m}-T_{0}}{erfc(K/2\sqrt{\alpha_{s}t})}$$

Substituting *A* and *B* from Equation (11) into Equation (10) yields the following expression:

(12) $$\begin{array}{l} T_{m}(x_{m},t)=T_{g}+\frac{T_{m}-T_{g}}{erf(K\beta /2\sqrt{\alpha_{m}})}erf(\frac{x_{m}(t)}{2\sqrt{\alpha_{m}t}})\\ T_{s}(x_{s},t)=T_{0}+\frac{T_{m}-T_{0}}{erfc(K/2\sqrt{\alpha_{s}})}erfc(\frac{x_{s}(t)}{2\sqrt{\alpha_{s}t}}) \end{array}$$

The theorem of the derivative of the error function can be provided by

(13) $$\frac{d}{dz}(erf(z))=\frac{2}{\sqrt{\pi }}e^{-z^{2}}$$

and then $T_{m}(x_{m,\;t})$ and $T_{s}(x_{s,\;t})$ can be derived according to Equation (13) as shown in Equation (14):

(14) $$\begin{array}{l} \frac{\partial T_{m}(x_{m},t)}{\partial x_{m}}=\frac{T_{m}-T_{g}}{\sqrt{\pi \alpha_{m}}erf(K\beta /2\sqrt{\alpha_{m}})}e^{-\frac{K^{2}\beta^{2}}{4\alpha_{m}}}\\ \frac{\partial T_{s}(x_{s},t)}{\partial x_{s}}=\frac{T_{m}-T_{0}}{\sqrt{\pi \alpha_{s}}erfc(K/2\sqrt{\alpha_{s}})}e^{-\frac{K^{2}}{4\alpha_{s}}} \end{array}$$

According to Equation (14), it can be found that the heating temperature $T_{g}$ has a significantly obvious effect on the molten zone, while has $T_{0}$ little effect. However, the opposite is true in the solid phase.

Substituting Equation (14) into Equation (5) yields Equation (15):

(15) $$k_{m}\frac{T_{m}-T_{g}}{\sqrt{\pi \alpha_{m}}erf(K\beta /2\sqrt{\alpha_{m}})}e^{-\frac{K^{2}\beta^{2}}{4\alpha_{m}}}-k_{s}\frac{T_{m}-T_{0}}{\sqrt{\pi \alpha_{s}}erfc(K/2\sqrt{\alpha_{s}})}e^{-\frac{K^{2}}{4\alpha_{s}}}=\frac{(1+\beta )LK\beta \rho_{m}}{2}-h_{o\&i}(T_{s}(x_{s},t)-T_{0})$$

For the convenience of solving, it is assumed that

(16) $$\lambda =\frac{K\beta }{2\sqrt{\alpha_{m}}}$$

Then Equation (15) can be transformed into the following form:

(17) $$\frac{e^{-\lambda_{o\&i}{\,}^{2}}}{erf(\lambda_{o\&i})}-\frac{k_{s}}{k_{m}}\sqrt{\frac{\alpha_{m}}{\alpha_{s}}}\frac{T_{m}-T_{0}}{T_{m}-T_{g}}\frac{e^{-\lambda_{o\&i}{\,}^{2}{\left(\rho_{m}/\rho_{s}\right)}^{2}(\alpha_{m}/\alpha_{s})}}{erfc(\lambda_{o\&i}\sqrt{\alpha_{m}/\alpha_{s}}\rho_{m}/\rho_{s})}=\frac{(1+\rho_{s}/\rho_{m})\sqrt{\pi }L}{C_{pm}(T_{m}-T_{g})}\lambda_{o\&i}-\frac{\sqrt{\pi \alpha_{m}}h_{o\&i}(T_{s}(x_{s},t)-T_{0})}{k_{m}(T_{m}-T_{g})}$$

where $C_{pm}$ denotes the specific heat of the HDPE pipe in the molten zone, KJ/kg·°C.

When heat convection is not considered, the distance from the solid–molten interface to the heated end can be predicted using the following equation:

(18) $$\frac{e^{-\lambda^{2}}}{erf(\lambda )}+\frac{k_{s}}{k_{m}}\sqrt{\frac{\alpha_{m}}{\alpha_{s}}}\frac{T_{m}-T_{0}}{T_{m}-T_{g}}\frac{e^{-\lambda^{2}(\alpha_{m}/\alpha_{s}){\left(\rho_{m}/\rho_{s}\right)}^{2}}}{erfc(\lambda \sqrt{\alpha_{m}/\alpha_{s}}\rho_{m}/\rho_{s})}=\frac{(1+\rho_{m}/\rho_{s})L\sqrt{\pi }}{C_{pm}(T_{m}-T_{g})}\lambda$$

Since Equations (17) and (18) are transcendental equations, the values of $\lambda_{o}$, $\lambda_{i}$, λ can be solved using the Newton–Raphson Method in Python 3.12.2 programming based on the data listed in Table 2, heating temperature, heating time, ambient temperature, and convective heat transfer coefficient. In the first equation of (12), let the left term of the equation equal the melting point of HDPE pipes. Thus, the relationship between the molten zone thickness and heating time at the outer surface, inner surface, and central line (the same as not considering heat convection) of HDPE pipes can be determined under fixed heating temperature, heating time, and ambient temperature.

(19) $$erf(\lambda_{o})=erf(\frac{x_{m}(t)}{2\sqrt{\alpha_{m}t}});erf(\lambda_{i})=erf(\frac{x_{m}(t)}{2\sqrt{\alpha_{m}t}});erf(\lambda )=erf(\frac{x_{m}(t)}{2\sqrt{\alpha_{m}t}})$$

Finally, Equation (20) can be simplified to obtain the following expression according to Equation (19):

(20) $$X_{o}=m\sqrt{t};X_{i}=n\sqrt{t};X_{c}=p\sqrt{t}$$

According to Equation (20), the AMZ thickness of HDPE pipe during butt fusion welding can be described as follows:

(21) $$AMZ=\frac{X_{o}+X_{i}+X_{c}}{3}$$

Due to the scope of application of Neumann’s solution for the melting of a semi-infinite region, this study considers only a short heating time, sufficiently long HDPE pipes, and ideal heat conduction, thereby avoiding the occurrence of multi-dimensional heat transfer effects. In future work, Neumann’s solution will need to be refined to accommodate more complex conditions, such as finite wall thickness, multi-dimensional heat transfer effects, and complex boundary conditions.

### 2.3. CFD Model for AMZ Thickness of HDPE Pipe

In this study, a two-dimensional CFD model was developed using FEA to validate the AMZ thickness of the HDPE pipe predicted by the MM model. The model features a nominal outside diameter (DN) of 200 mm, a standard dimensional ratio (SDR) of 17, a wall thickness of 11.76 mm, and a length of 30 mm to simulate phase change and heat transfer. In the CFD model, the AMZ thickness of the HDPE pipe is defined as the average molten zone thickness measured from multiple points on the solid–molten interface to the heated end, as shown in Figure 1b. The thermophysical material properties for the HDPE pipes were assigned based on Equation (1) and Table 1 and Table 2, ensuring that all essential properties such as density, thermal conductivity, specific heat capacity, melting point, and latent heat of fusion were included. The heated end was set to a heating temperature $T_{g}$ ranging from 190 °C to 350 °C in increments of 20 °C, while the right side was designated as a non-heated end with zero heat flux as shown in Figure 1b. The outer surface of HDPE pipes was exposed to the open environment with a convective heat transfer coefficient of 7.22 W/(m^2^∙°C), and the inner surface of HDPE pipes was in contact with an air interlayer with a convective heat transfer coefficient of 0.89 W/(m^2^∙°C). The convective heat transfer coefficients were set to zero for both the outer and inner surfaces of HDPE pipes in the CFD model when convection was disregarded, and radiation effects were not included in this model. The initial temperature was set to be 28 °C. The phase change temperature was set to 135 °C, with a 5 °C transition interval and a latent heat of 218 kJ/kg. The 5 °C transition interval was set to make the solid phase transition to the molten phase more smoothly to avoid non-convergence. The convergence criterion for the CFD model was set to 10^−6^ in FEA. The simulation was configured with a transient study, a total time of 180 s, and output intervals every 10 s. A default, extremely fine tetrahedral mesh was used, providing high resolution near the heated end. To obtain the AMZ thickness of the HDPE pipe in the CFD model, an isothermal line of 135 °C was set, and a series of coordinate points along the isothermal line were recorded as shown in Figure 1d,e. Since the heated end of the HDPE pipe was located at *x* = 0, the average of the x-coordinates of these points was calculated to be the AMZ thickness of HDPE pipes in the CFD model.

## 3. Results and Discussion

### 3.1. Parameter Solution of the Mathematical Model

HDPE pipes are widely used in various industrial applications due to their durability, flexibility, and corrosion resistance, with butt fusion welding being the most effective method for joining large-diameter, thick-walled HDPE pipes. The quality of welded joints in HDPE pipes is typically determined by optimal welding parameters, which ensure an appropriate molten zone thickness to achieve the desired bead-up height. Over the past decades, many researchers have investigated the thermal and mechanical behavior of HDPE pipes during butt fusion welding using experimental and numerical methods. However, limited research has focused on the theoretical analysis of how the molten zone thickness in HDPE pipes evolves with heating temperature and heating time during butt fusion welding. The purpose of this study is to develop an MM model to predict changes in the AMZ thickness of HDPE pipes as heating time progresses. Heating temperature, heating time, and heat convection between HDPE pipe surfaces and the surrounding environment were considered factors influencing the AMZ thickness during butt fusion welding. Section 2.2 provided a detailed description of the establishment of the MM model for predicting variations in the AMZ thickness of HDPE pipes under changes in heating temperature, heating time, and heat convection.

By solving transcendental Equations (17) and (18) using Python programming, the values of $\lambda_{o}$, $\lambda_{i}$, and λ can be obtained as shown in Table 3.

An analysis of Table 3 reveals that the values of $\lambda_{i}$ and λ exceed those of $\lambda_{o}$ and remain very close to each other. A larger lambda value indicates better thermal insulation performance of HDPE pipes during butt fusion welding. Conversely, a smaller lambda value indicates a higher heat dissipation capability. These values of $\lambda_{o}$, $\lambda_{i}$, and λ from Table 3 are then substituted into Equation (19) to determine the values of *m*, *n*, and *p* in Equation (20), as shown in Table 4.

From Table 4, it is observed that *m*, *n*, and *p* follow the same trend as $\lambda_{o}$, $\lambda_{i}$, and λ. The molten zone thickness at the outer surface, central line, and inner surface of HDPE pipes can be determined by substituting *m*, *n*, and *p* from Table 4 into Equation (20). Larger values indicate a greater molten zone thickness during butt fusion welding at the same heating time and temperature. The AMZ thickness in the MM model can then be determined using Equation (21). To verify the reliability of the MM model results, this study also analyzed the AMZ thickness of HDPE pipes using a two-dimensional CFD model using FEA under the same boundary conditions and material properties. The 135 °C isothermal contours are shown in Figure 1d,e, corresponding to cases where heat convection between HDPE pipe surfaces and the environment is included and neglected, respectively.

### 3.2. Effect of Heating Temperature on AMZ Thickness

**Figure 2:**
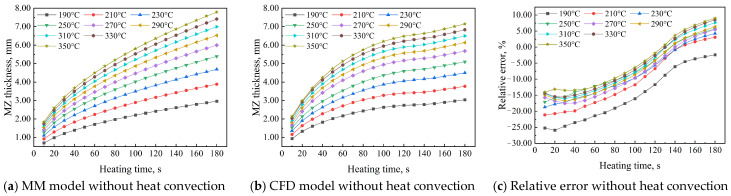
AMZ thickness of HDPE pipe in MM and CFD models without heat convection.

Heating temperature in butt fusion welding is a key parameter that primarily determines molten zone thickness and. This study investigated the effects of heating temperatures ranging from 190 °C to 350 °C in 20 °C increments and heating times from 10 s to 180 s (in 10 s increments) on the AMZ thickness in HDPE pipes using Equations (20) and (21) from the MM and CFD models. Figure 2 compares the AMZ thickness predictions from the MM and CFD models without heat convection, along with their relative error, for heating temperatures from 190 °C to 350 °C at a heating time of 180 s. Both models show nonlinear growth in AMZ thickness over heating time, with higher temperatures leading to thicker molten zones in HDPE pipes. The trends in Figure 2a,b reveal two distinct AMZ thickness change rate behaviors. Initially, the rate decreases with increasing heating time, indicating a slowdown in AMZ thickness change due to thermal stabilization over extended durations. Conversely, the rate increases with rising heating temperatures, indicating that higher thermal energy accelerates melting and enhances the AMZ thickness change rate. Notably, in the CFD model, an exception occurs between 140 and 180 s, where the AMZ thickness change rate increases across all heating temperatures, deviating from the general trend. This suggests that additional heat accumulation or a nonlinear phase transition may affect melting dynamics during this period. These observations highlight the combined influence of time-dependent heat diffusion and temperature-dependent melting dynamics on AMZ thickness evolution. During the initial phase of heat conduction in butt fusion welding, heat rapidly diffuses from the heated end, quickly melting the surface and creating a steep temperature gradient that drives the molten zone to expand inward. In a short time, additional heat input and minimal dissipation rapidly increase molten zone thickness. As heating time progresses, the temperature gradient decreases, the conductive path lengthens, and the molten zone thickness growth slows. In the MM model without considering heat convection, the AMZ thickness is 2.965 mm at 190 °C and 7.793 mm at 350 °C for a heating time of 180 s. In contrast, the CFD model without heat convection predicts an AMZ thickness of 3.038 mm at 190 °C and 7.150 mm at 350 °C for the same heating duration. Figure 2c illustrates the variation in relative error between the MM and CFD models. All curves show an upward trend with increasing heating time, though the rate of increase varies with heating temperature. When comparing AMZ thickness between the MM and CFD models at a heating time of 180 s, the absolute error ranges from −0.073 mm to 0.643 mm, and the relative error ranges from −2.398% to 8.992%, assuming no heat convection between the HDPE pipe surfaces and the open environment.

### 3.3. Effect of Heat Convection on AMZ Thickness

**Figure 3:**
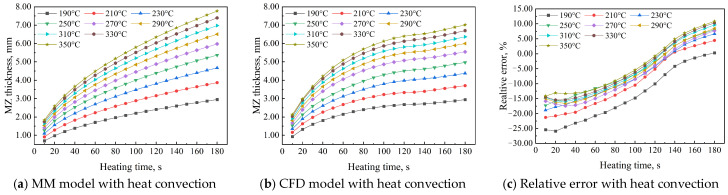
AMZ thickness of HDPE pipe in MM and CFD models with heat convection.

To achieve high-quality welded joints in HDPE pipes, the developed MM model can be used to determine optimal welding parameters, minimizing experimental waste and computational costs while significantly improving welding efficiency. Additionally, environmental conditions must be considered during butt fusion welding. This study examined the effects of heat convection between surfaces of HDPE pipes and the open environment on AMZ thickness using MM and CFD models. During butt fusion welding of HDPE pipes, heat exchange with the open environment affects the outer surface, while heat exchange within the air interlayer affects the inner surface. The open environment enables the outer surface to fully engage in heat convection with the surrounding air, while the inner surface, adjacent to an air interlayer, acts as an insulating layer, reducing heat transfer between the pipe’s interior and the environment and enhancing thermal insulation. This can be proved by the convective heat transfer coefficient of 7.22 W/(m^2^∙°C) between the outer surface of the HDPE pipes and the open environment and the convective heat transfer coefficient of 0.89 W/(m^2^∙°C) between the inner surface of the HDPE pipes and air interlayer. Therefore, heat dissipation is strongest at the outer surface, moderate at the inner surface, and weakest at the central line. As a result, the molten zone thickness is smallest at the outer surface and largest at the central line of the HDPE pipe. The AMZ thickness of HDPE pipes in both the MM and CFD models, considering heat convection between the surfaces of HDPE pipes and the open environment, along with the absolute and relative errors between the models, exhibits similar variations to those in the previous analysis, as shown in Figure 3. In the MM model, the AMZ thickness is 2.953 mm at 190 °C and 7.774 mm at 350 °C for a heating time of 180 s. In contrast, in the CFD model without heat convection, it is 2.944 mm at 190 °C and 7.017 mm at 350 °C for the same duration. The absolute error between the MM and CFD models ranges from 0.08 mm to 0.760 mm, while the relative error ranges from 0.280% to 10.830%.

### 3.4. Comparison of the Effects on AMZ Thickness with and Without Heat Convection

**Figure 4:**
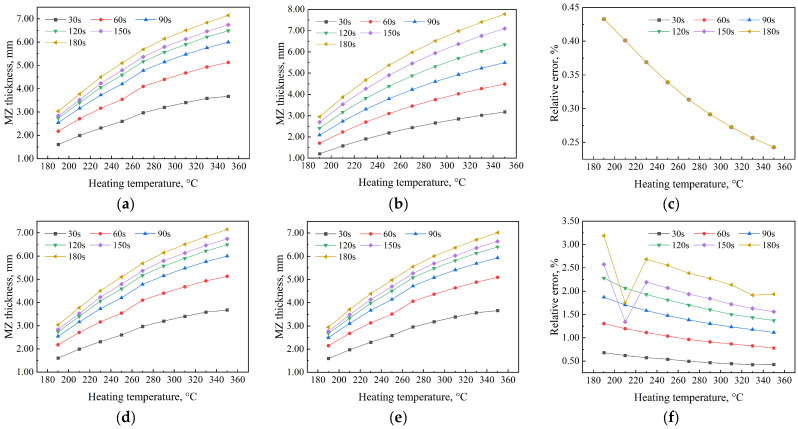
Comparison of the effects of heat convection on the AMZ thickness of HDPE pipe. (**a**) MM model without heat convection; (**b**) MM model with heat convection; (**c**) relative error with and without heat convection; (**d**) CFD model without heat convection; (**e**) CFD model with heat convection; (**f**) relative error with and without heat convection.

Butt fusion welding of HDPE pipes occurs in a real physical environment, where environmental factors such as air velocity and dynamic viscosity inevitably affect AMZ thickness. Typically, these environmental factors are used in determining the relevant convection heat transfer coefficients between the surfaces of HDPE pipes and the open environment. In this section, the AMZ thickness of HDPE pipes in both the MM and CFD models was determined with and without thermal convection effects. Figure 4 illustrates the variation in the AMZ thickness of HDPE pipes with heating temperature using the MM and CFD models. The analysis examines the AMZ thickness of HDPE pipes for heating times of 30, 60, 90, 120, 150, and 180 s. According to Figure 4a–c, the AMZ thickness of HDPE pipes, with and without heat convection, shows a high level of agreement. At 190 °C, the relative error, both with and without heat convection, is highest at 0.433%, while at 350 °C, it is lowest at 0.243%. Additionally, the relative error curves for all heating times overlap due to the stability and assumptions of the MM model. In Figure 4d–f, a comparison of the relative error in AMZ thickness between the CFD and MM models shows that, while both values are small, noticeable differences remain. Regardless of whether heat convection between the HDPE pipe surface and the environment is considered, the AMZ thickness in the MM model increases consistently at a uniform rate. However, in the CFD model, the increase is neither consistent nor uniform, as seen by comparing Figure 4d,e with Figure 4a,b. It can be observed that shorter heating times result in a greater increase in AMZ thickness, aligning perfectly with Figure 2a,b and Figure 3a,b. In the CFD model, at the same heating temperature, relative errors in AMZ thickness vary with heating time, showing significant differences from the MM model results. This occurs because the MM model relies on assumptions and theoretical formulations, ignoring multi-physics coupling effects, whereas the CFD model accounts for them. A comparison of Figure 4f and Figure 4c shows that the relative error in the former is larger than in the latter and varies with heating time. The largest relative error of the AMZ thickness of HDPE pipes occurs at a temperature of 190 °C and a heating time of 180 s, with a value of 3.189%, while the smallest relative error of the AMZ thickness of HDPE pipes occurs at a temperature of 350 °C and a heating time of 30 s. It is worth noting that Figure 4f shows distinct variations in the CFD model, where nonlinear thermophysical parameters and two-dimensional heat conduction introduce more significant differences. An abrupt change in relative error occurs at 210 °C when the heating time is 150 s and 180 s. Under lower heating temperatures (190 °C and 210 °C), the CFD model predicts a smaller molten zone thickness, making thermal convection heat dissipation more pronounced. Consequently, the relative error between cases with and without thermal convection is significantly higher in this temperature range. As the heating temperature increases, the molten zone thickness grows, reducing the relative influence of thermal convection and leading to a gradual decrease in relative error until stabilization. These findings highlight the need to consider environmental effects on molten zone thickness in real welding processes, particularly at lower heating temperatures.

This study, using both the MM and CFD models, compared the effects of neglecting and incorporating heat convection between HDPE pipe surfaces and the open environment on AMZ thickness, as shown in Figure 4. The results indicate that the relative error in AMZ thickness ranges from 0.243% to 0.433% in the MM model and from 1.751% to 3.189% in the CFD model.

### 3.5. Analysis of Potential Errors Affecting AMZ Thickness in MM and CFD Models

This section thoroughly investigates the potential error sources affecting AMZ thickness in both MM and CFD models. Neumann’s solution for the melting of a semi-infinite region was employed for the development of the MM model. However, due to its scope of application, the three-dimensional heat transfer effects present in the actual welding process were ignored, particularly at the edges and corners of the pipe. This simplification may result in inaccuracies, either underestimating or overestimating the AMZ thickness in HDPE pipes. In addition, HDPE pipes exhibit viscoelastic behavior, with mechanical properties that depend on both heating temperature and heating time. The omission of viscoelastic behavior in the MM model may reduce the accuracy of AMZ thickness predictions in HDPE pipes during butt fusion welding. Furthermore, the MM model assumes uniform and constant thermal properties for HDPE pipes in both the solid and molten phases. However, in reality, these properties vary significantly with temperature and phase transitions, especially at high temperatures, potentially leading to inaccuracies in AMZ thickness predictions. It must be noted that neglecting welding pressure and assuming a constant convective heat transfer coefficient may further contribute to errors in the AMZ thickness of HDPE pipes obtained from the MM model. In the CFD model, some error sources are similar to those in the MM model. However, additional factors affecting the accuracy of AMZ thickness prediction include the resolution of the computational mesh, which can influence numerical diffusion and boundary layer representation, and the choice of the transition temperature interval in the solid–liquid phase, which impacts the accuracy of phase change modeling. To minimize potential errors and enhance the accuracy of AMZ thickness predictions in HDPE pipes, future studies should refine both the MM and CFD models by addressing the identified error sources. Specifically, improvements should include incorporating viscoelastic behavior into the MM model, refining temperature-dependent thermal property variations, enhancing computational mesh resolution, optimizing phase transition modeling, and considering the effects of welding pressure and convective heat transfer variations.

## 4. Conclusions

This study successfully developed an MM model to predict AMZ thickness in HDPE pipes during butt fusion welding, achieving high accuracy and efficiency, as validated by comparisons with CFD model results. The MM model provided reliable predictions, with relative errors ranging from 0.280% to 10.830% with heat convection and −2.398% to 8.992% without it, across the specified heating temperature range and a heating time of 180 s. Importantly, the effect of heat convection on AMZ thickness was identified, with relative errors ranging from 0.243% to 0.433% in the MM model and 1.751% to 3.189% in the CFD model, affirming the robustness of the developed MM model. This study contributes to understanding how molten zone thickness in HDPE pipes evolves with heating temperature and time during butt fusion welding from a theoretical perspective, to some extent. By enhancing both the theoretical understanding and practical control of welding parameters, this study improves the accuracy and efficiency of butt fusion welding in HDPE pipes. The developed MM model has significant industrial potential, optimizing welding procedures by reducing reliance on extensive experimental trials and computational resources. However, this study acknowledges certain limitations, as discussed in Section 3.5. These limitations may affect model precision under complex real-world conditions. Moreover, there is a lack of experimental validation for the results obtained from the MM and CFD models. Future research should take the limitations listed in this study into consideration for the improvement of MM and CFD models, explore multi-physics coupling effects, and extend model validation to different pipe dimensions and more complex environmental interactions. These advancements will enhance the reliability, applicability, and quality of butt fusion welding in HDPE pipes.

## Figures and Tables

**Table 1:** The thermophysical material properties of HDPE pipes in response to temperature change [24].

Temperature °C	Thermal Conductivity $\frac{W}{m\cdot {}^{\circ}C}$	$\mathrm{Isobaric}\;\mathrm{Heat}\;\mathrm{Capacity}\;\frac{\mathrm{KJ}}{\mathrm{kg}\cdot {}^{\circ}C}$	Enthalpy $\frac{\mathrm{KJ}}{\mathrm{kg}}$
0	0.445	1.83	0
50	0.405	2.05	95
100	0.340	2.87	200
125	0.300	5.56	318
132	0.268	15.51	485
150	0.265	2.65	535
200	0.264	2.67	700
250	0.263	3.03	900

**Table 2:** The thermophysical material properties of HDPE pipes in the MM model phase [25].

	Density $\frac{\mathrm{kg}}{m^{3}}$	Isobaric Heat Capacity $\frac{\mathrm{KJ}}{\mathrm{kg}\cdot {}^{\circ}C}$	Thermal Conductivity $\frac{W}{m\cdot {}^{\circ}C}$	Thermal Diffusivity $\frac{m^{2}}{s}$
Solid phase	980	2.31	0.49	2.16 × 10^−7^
Melting phase	780	2.51	0.26	1.18 × 10^−7^
Melting temperature, °C		135	Latent heat, $\frac{\mathrm{KJ}}{\mathrm{kg}}$	218

**Table 3:** The values of $\lambda_{o}$, $\lambda_{i}$, and λ in the transcendental Equations (17) and (18).

	190 °C	210 °C	230 °C	250 °C	270 °C	290 °C	310 °C	330 °C	350 °C
$\lambda_{o}$	0.318	0.418	0.505	0.580	0.646	0.704	0.755	0.800	0.841
$\lambda_{i}$	0.322	0.422	0.509	0.585	0.651	0.709	0.760	0.805	0.846
λ	0.322	0.422	0.510	0.585	0.651	0.709	0.760	0.806	0.846

**Table 4:** The values of *m*, *n*, and *p*, ×10^−4^, for Equation (20).

	190 °C	210 °C	230 °C	250 °C	270 °C	290 °C	310 °C	330 °C	350 °C
*m*	2.185	2.866	3.463	3.981	4.433	4.829	5.179	5.491	5.771
*n*	2.207	2.893	3.493	4.013	4.466	4.862	5.212	5.524	5.804
*p*	2.210	2.897	3.497	4.018	4.470	4.867	5.217	5.529	5.809

## Data Availability

The original contributions presented in this study are included in the article. Further inquiries can be directed to the corresponding author.
